# Impact of gut probiotic metabolites on phenylketonuria

**DOI:** 10.3389/fcimb.2025.1682110

**Published:** 2025-10-13

**Authors:** Anjana Kalla Veedu, Sujithra Vijayakumar, Hannah A. Joseph, John Thomas

**Affiliations:** Centre for Nanobiotechnology, Vellore Institute of Technology (VIT), Vellore, Tamil Nadu, India

**Keywords:** phenylketonuria, metabolic disorders, human, gut probiotics, amino acids, phenylalanine, mechanism, tyrosine

## Abstract

Phenylketonuria is an unusual inherited metabolic disease induced by mutations of the phenylalanine hydroxylase gene, resulting in phenylalanine accumulation. The current treatments only focus on restricting lifelong dietary intake of phenylalanine, posing a significant challenge to concordance and living standards. Emerging evidence on phenylketonuria disorders underscores the gut microbiome involving probiotics as a key mediator of host metabolic processes. This review encompasses the insights into the pathophysiology of phenylketonuria, gut probiotics, the amino acid metabolism of phenylalanine, the mechanism of action of probiotics, and the therapeutic potential of the treatments available.

## 1 Introduction

Phenylketonuria, otherwise known as a deficiency of phenylalanine hydroxylase (PAH), is an autosomal recessive genetic disorder. It is often distinguished by the inability to metabolize the phenylalanine amino acid (AA) resulting from PAH inadequacy. PAH is crucial for the conversion of tyrosine from phenylalanine, an important essential AA for various functions in humans. The deficiency or absence of PAH leads to the aggregation of phenylalanine in the blood and brain, inducing various neurological and metabolic disorders. Whereas higher concentrations of phenylalanine induce brain dysfunction. In the absence of treatment, the dysfunction leads to acute intellectual impairment, epilepsy, and behavioral complications (van Spronsen et al., 2021; Russo et al., 2025).

The prevalence of Phenylketonuria is identified worldwide in many countries, and its variation is larger when compared to its prevalence. In the United States, the overall incidence of Phenylketonuria was about 1/15,000. It was even greater for Caucasians and native Americans. Whereas it was lower for African American, Asian, and Hispanic populations. In countries such as Turkey, it was found to be as high as 1/4000 live births (Phenylketonuria, 2023). The prevalence of Phenylketonuria in the newborn screening coverage done in Brazil was identified with an annual incidence of 4–8 per/100,000 live births. Further, a record of 7615 patients was registered in their health systems (Vargas et al., 2025). Based on the reports of a French database on January 1, 2018, 3549 patients were reported with Phenylketonuria (Arnoux et al., 2024).

Probiotics or “live micro-organisms”, when consumed in an appropriate dose, have an impact on their host. Important functions like metabolic, barrier effects, and trophic functions rely on these bacterial communities. Dysbiosis in them has a negative impact on human health as well. The interactions between the human gut system, nutrients, microbes, and host cells significantly contribute to the gut homeostasis and development of the host. So, the application of probiotics has gained importance in disease treatment (Butel, 2014). Gut microbes play a pivotal part in metabolic processing in humans, and their alteration may lead to various metabolic disorders. In recent times, novel treatments using engineered probiotics for handling various metabolic disorders are being developed (Barati et al., 2024).

The primary treatment for phenylketonuria disease is the restriction of Phenylalanine from the patient’s diet, augmented with L-AA. Trillions of gut-associated bacteria or gut microbiota are present in healthy humans. The gut microbiota constitution can be modulated by various environmental and external factors, including diets and antibiotic exposure. The nutrition in the food of Phenylketonuria patients affects the host physiology as well as the gut microbiome environment. A study was conducted to characterize the gut microbiota of Phenylketonuria patients and healthy individuals using next-generation sequencing. It revealed the reduction of certain bacterial species from healthy individuals, indicating the significant differences in the metabolism of glucose and amino acids (De Oliveira et al., 2016).

This review comprises the pathophysiology of Phenylketonuria, its treatment methods, the exclusive role of probiotics, and their role in disease management. Further, the mechanism of action of probiotics, the limitations, and challenges in treating this metabolic disorder are discussed in detail, including an update on the probiotics and Phenylketonuria.

## 2 Pathophysiology of phenylketonuria

**Figure 1:**
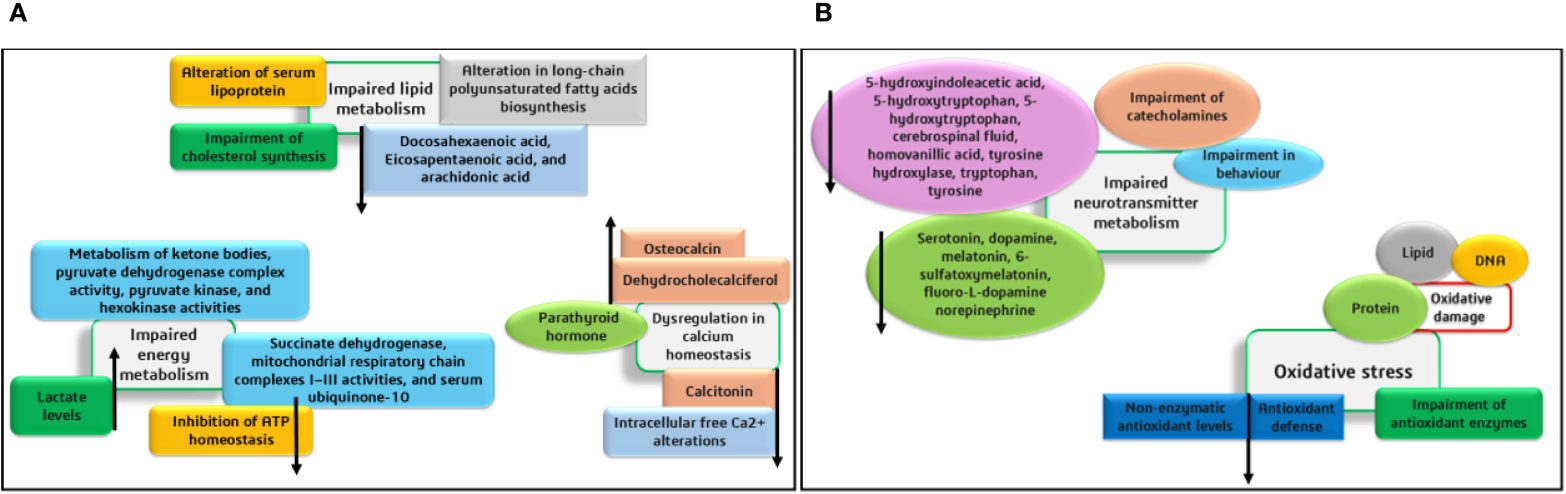
**(A, B)** Impacts of various metabolic alterations caused by phenylketonuria.

The organs and body fluids of Phenylketonuria patients are affected by the phenylalanine accumulation and its metabolites due to PAH deficiency. In the kidney and liver, the major phenylalanine metabolic pathway requires the hydroxylation of PAH by Tyrosine, and other alternative metabolic pathways are more trivial. The Phenylketonuria patients show intense neurological damage in the regions of the corpus callosum and striatum. Alterations in cortical regions and hypomyelination further lead to intellectual shortfall and neurodegeneration. In animal and human systems, metabolic changes, including oxidative stress, defective protein and neurotransmitter synthesis, and mitochondrial disorders were identified. The impacts of various metabolic alterations are depicted in **Figures 1A, B**. Further, the mechanisms involved in the disordered cerebral metabolisms and cognitive functional developments have not been completely unraveled (Schuck et al., 2015; de Groot et al., 2010).

Studies also state that hypothetically, the main factor behind the pathophysiological mechanism of cognitive disorders is the reduction of cerebral neurotransmitters (like dopamine and serotonin) caused by reduced availability of the AAs (Tyrosine and tryptophan). Additionally, in the presence of higher phenylalanine levels, the decrease in protein synthesis occurs due to the irregular uptake of non-phenylalanine large neutral AAs by the brain. The findings of researchers suggest that the major symptoms of this disease are mostly found in the brain, and the exact mechanism of pathophysiology is yet to be explored for a detailed understanding (Schuck et al., 2015; de Groot et al., 2010). Recent findings suggest that PAH is crucial for catalyzing the hydroxylation of phenylalanine to tyrosine, and this process includes the cofactor BH4 (rate-limiting step). During this process, BH4 is converted to quinonoid dihydrobiopterin (qBH2- oxidized form). The PAH enzyme facilitates the irreversible conversion of phenylalanine to tyrosine. The Cofactor BH4 is synthesized from guanosine triphosphate in different tissues, including the liver, and is also recycled after the hydroxylation of phenylalanine through enzyme-based catalytic reduction. The deficiency of PAH leads to phenylalanine accumulation in all tissues and also causes relative tyrosine deficiency (Elhawary et al., 2022).

From a genetic perspective, Phenylketonuria is an inborn defect of phenylalanine metabolism due to pathogenic variants in the PAH gene. Further, genetic studies reveal that an individual with PKU receives a defective gene from their parents (both), and the gene located on chromosome 12q23.2 (90 kb) plays a role in numerous mutations in the PAH gene (autosomal recessive pattern). These mutations induce a reduction in PAH activity or a complete deficiency of the PAH enzyme. More than 1000 PAH gene variants have been reported, and their effects vary with the residual range of PAH activity in Phenylketonuria patients (Blau, 2016; van Spronsen et al., 2021; Russo et al., 2025; Elhawary et al., 2022).

## 3 Role of gut microbiota in amino acid metabolism – phenylalanine

Phenylketonuria is a metabolic disorder caused by a deficiency of the hepatic enzyme PAH, resulting in high levels of the AA phenylalanine in the blood and brain. This leads to severe cognitive and psychological deficits, which can be partially prevented by dietary treatment. The behavioral pattern of Phenylketonuria patients can be linked to the gut-microbiome-brain axis, as diet plays a crucial role in modulating gut microbiome composition (van der Goot et al., 2022). The Gut microbiota and the central nervous system are related through major pathways such as the intestinal-mucosal barrier way, immune system route, gut-microbiome metabolic pathway, neuroanatomical pathway, the neuroendocrine-hypothalamic–pituitary–adrenal axis pathway, and the blood-brain barrier. In consideration of Phenylketonuria, the diet plays a significant role in the gut-brain axis, as it affects the key intermediaries involved in the communication pathway. The microbes that produce metabolites like short-chain fatty acids, Gamma-aminobutyric acid, threonine, serotonin, glutamate, carnosine, Phenylalanine, aspartic acid, alanine, lysine, tyrosine, glycine, gut hormones/incretins, and ammonia may influence brain function or vice versa. Furthermore, gut microbiota might regulate the integrity of both the gut and the blood-brain barrier. This might lead to improper nutrient absorption (Mucosal surface) (Verduci et al., 2020).

A phenylalanine-restricted diet provided with additional L-AAs is the core treatment for this disease, and in a study conducted by De Oliveira et al., 2016, the gut microbiota of 8 Phenylketonuria patients with a phenylalanine-restricted diet and 10 healthy individuals were compared. The result indicated that *Bacteroidetes* and *Firmicutes* sp., were the most dominant phyla in both groups. Phenylketonuria patients showed reduced abundance of *Odoribacter* Sp., *Coprococcus* Sp., *Ruminococcus* Sp.*, Erysipelotrichaceae*, *Lachnospiraceae* families, *Clostridiaceae*, and *Clostridiales* class, *Dorea*, *Lachnospira*, and *Veillonella* genera, and enrichment of *Peptostreptococcaceae*, *Prevotella*, and *Akkermansia* (De Oliveira et al., 2016).

Recent studies have shown that dietary phenylalanine, upon reaching the colon, is metabolized by specific gut microbes through enzymatic pathways involving phenylpyruvate decarboxylase and phenylpyruvate-ferredoxin oxidoreductase. These microbial actions lead to the production of phenylacetic acid, which is subsequently conjugated in the liver with glutamine to form phenylacetyl glutamine (PAGln). PAGln is not just a metabolic byproduct—it also functions as a bioactive compound linked to thrombosis, cardiovascular disease, and neuroinflammation. Elevated levels of PAGln have been linked to gut dysbiosis and chronic diseases involving the heart, kidney, and brain. This highlights that the microbial transformation of AAs can extend beyond the gut, influencing systemic health (Krishnamoorthy et al., 2024).

Phenylalanine is a significant aromatic AA in Phenylketonuria disease. Tyrosine is synthesized from phenylalanine hydroxylation, and tyrosine hydroxylase is the critical enzyme responsible for converting tyrosine into catecholamines like norepinephrine, epinephrine, and dopamine. Phenylalanine and tyrosine are mediated via the fermentation process by gut microbes into phenolic compounds. The two phenolic derivatives, p-cresol sulfate and p-cresol, are absent in germ-free mice, indicating the impact of the gut microbes in the production of both compounds. Bacterial species like *Proteus vulgaris*, *Lactobacillaceae*, *Clostridium difficile*, and *C. scatalogenes* are engaged in the formation of p-cresol from tyrosine. Furthermore, the relative abundance of the two bacterial metabolites p-cresol and phenylacetylglutamate, which originated from AAs (phenylalanine and tyrosine) metabolism, is greater in persons with multiple sclerosis compared to controls (Rebeaud et al., 2022). These studies correlate the role of gut microbes in altering the metabolic processes in humans.

## 4 Mechanism of action of probiotics

Probiotics have been used as food and feed for humans and animals over the years, which has helped to keep the gut healthy and balanced (Sakandar and Zhang, 2021; Xavier-Santos et al., 2022). Probiotics play a prominent role in gut microbiota, metabolic processes, and immune responses (Pires et al., 2024). It has been shown that the correlation between probiotics and gut microbiota is closely related to systemic inflammation and metabolic homeostasis (Chandrasekaran et al., 2024). Although the benefits of probiotics have been understood, the mechanism through which they function is still under investigation (Gul and Durante-Mangoni, 2024).

**Figure 2:**
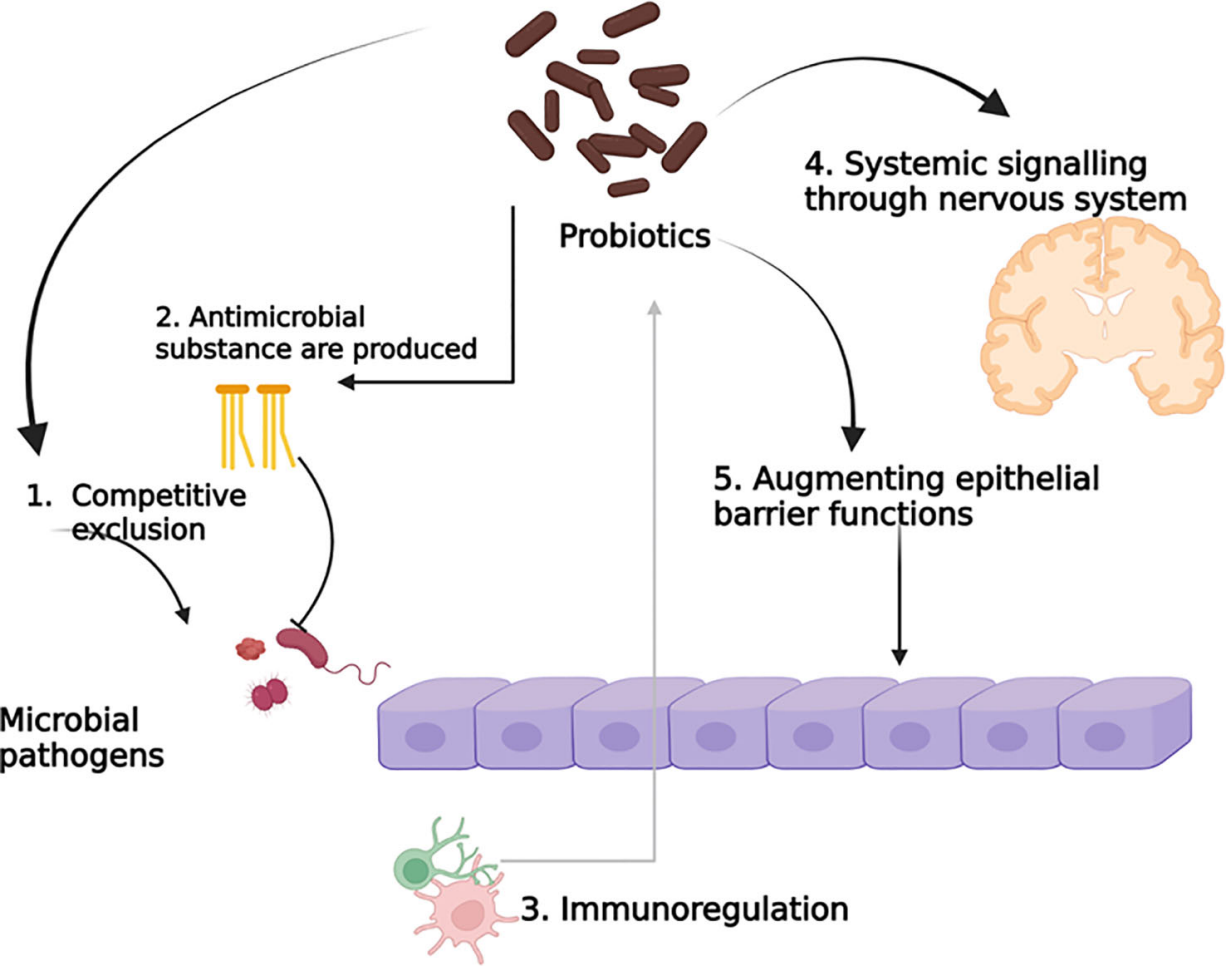
Potential probiotic mechanism of action.

The mode of action of probiotics mainly focuses on metabolite production, such as vitamins, 1,4-dihydroxy-2-naphtoic acid, short-chain fatty acids, a bifidogenic and anti-inflammatory product (Le Barz et al., 2015). However, the probiotic can change the gut microbiota directly, or the gut microbiota randomly changes with probiotic treatment, as the changes can happen in both directions, and as a result, they are connected to each other. However, it remains unclear whether the two-way mechanism and interactions are dependent or independent. Probiotics are associated with cell components such as peptidoglycans, polysaccharides, teichoic acids, deoxyribonucleic acid, cell-surface-bound and secretory proteins, bacteriocins, polyphosphate, and fatty acids, which can obstruct interactions. Phenylalanine ammonia-lyase has a potential therapeutic effect, which was obtained from bacteria in Phenylketonuria when administered orally through *Escherichia coli* Nissle 1917 and *Lactobacillus reuteri* (Durrer et al., 2017; Isabella et al., 2018; Puurunen et al., 2021). The potential probiotic mechanism of action is depicted in **Figure 2**.

## 5 Phenylalanine metabolic mechanisms in phenylketonuria

Phenylketonuria is one of the genetic disorders that is seen in approximately 1 in 23,930 newborns (Hillert et al., 2020). It is an autosomal recessive condition, which in turn means both parents should carry and pass the recessive gene (Bobrova et al., 2025). In chromosome 12q23.2, the PAH gene is located, which codes for the PAH enzyme involved in the metabolic pathway of phenylketonuria (Blau et al., 2010). The catalysis of L-phenylalanine to L-tyrosine by para-hydroxylation of the aromatic side chains was done by PAH (Flydal and Martinez, 2013). PAH is associated with the family of aromatic amino acid hydroxylases. Phenylalanine is an essential amino acid, whereas tyrosine can be synthesized by the hydroxylation of phenylalanine. When PAH is low in the body, it results in the excess formation of phenylalanine, leading to hyperphenylalaninemia and Phenylketonuria (Williams et al., 2008). Tetrahydrobiopterin (BH4) can bind to the domain for catalysis of PAH, and it acts as the cofactor for the reaction. The exact mechanism of phenylalanine on brain damage is still unclear, but the impact on the central nervous system depends on the presence of phenylalanine in the brain, with younger, developing brains being more vulnerable (van Spronsen et al., 2009; Hawks et al., 2019; Romani et al., 2017).

Over the last decades, new treatments have been introduced to modify microbiota. Probiotic integration is also highlighted in this treatment process. The microbiota changes the microbial population, alters their composition, and affects the functionality of the microbes. Therefore, studies show that therapies can help treat metabolic disorders (de Groot et al., 2017; Le Barz et al., 2015). Isabella et al. (2018) formulated a synthetic strain of *E. coli* that can produce some enzymes that can break down the amino acid phenylalanine, which can even work in low oxygen conditions (Isabella et al., 2018). Recent research has examined how the Phenylketonuria treatment diet and phenylalanine affect children, showing a significant difference in their gut microbiota between Phenylketonuria individuals and non-Phenylketonuria individuals (Bassanini et al., 2019; De Oliveira et al., 2016).

Genetically modified probiotics, which are developed as live biotherapeutic agents, represent advanced and targeted alternative treatment methods for treating Phenylketonuria (de Oliveira Filho et al., 2022). Clinical development of therapeutics will have to adhere to strict regulatory controls and cannot be scaled in the same way as other products (Charbonneau et al., 2020). In Phenylketonuria patients, microbial community diversity is lowered, causing an imbalanced gut microbiome (dysbiosis) (Mosca et al., 2016), which means that some beneficial bacterial species are missing or reduced partially (Verduci et al., 2018; Mancilla et al., 2021; Pinheiro de Oliveira et al., 2016). The gut microbiota imbalance of Phenylketonuria patients is influenced by the nutrient intake in their diet. They consume higher glycemic index and glycemic load foods when compared to patients with mild hyperphenylalaninemia (Verduci et al., 2020, 2018). *In vitro* experiments show that in Phenylketonuria, some chemicals are formed from the breakdown of Phenylalanine and form phenylpropionic acid, phenyllactic acid, and phenylacetic acid, which interfere with the antioxidant enzymes. The accumulated substance affects the function and activity of these cells, which protects the body from oxidative damage. Phenyllactic acid and phenylacetic acid increased the activity of superoxide dismutase in animals, while phenylpropionic acid decreased the glucose-6-phosphate dehydrogenase in brain tissues (Moraes et al., 2013; Rosa et al., 2012).

## 6 Therapeutic potential

Initially, the most widely used therapy for Phenylketonuria patients was a blend of protein and Phenylalanine-restricted nutritional food. In 2007, the Food and Drug Administration approved sapropterin dihydrochloride, the first pharmacological agent for phenylketonuria, which is a synthetic version of the naturally occurring cofactor tetrahydrobiopterin (BH4). BH4 is essential for PAH activity, and any defects in its synthetic pathway can result in PAH deficiency and elevated Phenylalanine concentrations. Pegvaliase, endorsed in 2018, was a medical intervention for Phenylketonuria patients. It is a novel substitutive enzyme therapy containing pegylated phenylalanine ammonia lyase, especially for patients unresponsive to sapropterin. Administered through subcutaneous injection daily, it converts Phenylalanine into ammonia and trans-cinnamic acid. Phenylketonuria patients aged 18 and older in the United States were approved for its usage. Clinical trials showed that individuals administered pegvaliase showed a much greater reduction of phenylalanine levels when compared to those who followed diet-based treatments or a blend of diet and sapropterin. After a treatment of 2 years, 61% and 51% of treated people reached phenylalanine levels of ≤360 and ≤120 μmol/L (blood), respectively. However, pegvaliase is associated with adverse effects ranging from benign reactions in injection regions to anaphylaxis. Consequently, all patients, pharmacies, and practitioners are obliged to enroll in a risk analysis and mitigation framework program before formerly initiating the therapy (Shrestha et al., 2025).

In a study conducted by Williams et al. (2025), Sepiapterin, a natural precursor of BH4, was developed as a treatment for children and adults with Phenylketonuria. Sepiapterin is safe and effective in lowering the blood phenylalanine levels in responsive individuals. The phase III APHENITY trial demonstrated that sepiapterin significantly lowered elevated phenylalanine levels in responsive children and adults with Phenylketonuria. Ongoing long-term studies aim to assess its effects on neurocognitive function, overall health status, nutritional outcomes, and quality of life in treated individuals. **Table 1** describes the different therapies available for phenylketonuria, their efficacy mechanism, and side effects.

**Table 1:** Different therapies available for phenylketonuria, their efficacy mechanism, and side effects.

S. no	Therapy	Mechanism	Target group	Efficacy	Side effects	Approval status	Reference
1	Low Phenylalanine Diet	Restricts Phenylalanine intake to prevent toxic buildup; supplemented with Phenylalanine-free AA mixture	All Phenylketonuria patients, especially infants and children	Effective in preventing intellectual disability and maternal Phenylketonuria syndrome	Difficult long-term adherence; neuropsychiatric symptoms with non-compliance	Standard initial therapy	Pey et al., 2008
2	Large Neutral AAs	Competes with transportation Phenylalanine across the blood-brain barrier, reducing brain Phenylalanine.	Adolescents and adults with poor diet adherence	May reduce brain Phenylalanine temporarily	Short-term effect: does not lower blood Phenylalanine	Not Food and Drug Administration approved as a monotherapy	Al Hafid and Christodoulou, 2015
3	Sapropterin Dihydrochloride	Synthetic BH4 cofactor stabilizes/rescues mutant PAH enzyme	BH4-responsive patients (mild/moderate Phenylketonuria)	20-56% show ≥30%reduction in Phenylalanine levels	Diarrhea, cough, Phenylalanine below normal range in children; limited pregnancy safety data	Food and Drug Administration-approved (2007) as an adjunct to diet	Al Hafid and Christodoulou, 2015
4	Pharmacological Chaperons (e.g., Compound III and IV)	Stabilize misfolded PAH protein, increase activity, and promote tetramer formation	*In vitro*/*in vivo* tested in mice and cell lines with mutant PAH	Increased PAH activity and protein levels in cells and mouse liver	Early research stage; safety/effectiveness in humans unknown	Preclinical stage (research only)	Nulmans et al., 2025
5	Phenylalanine Ammonia Lyase/Pegvaliase	Enzyme substitution therapy converting Phenylalanine – trans-cinnamic acid+ammonia	Adults with uncontrolled Phenylalanine levels on conventional therapy	Up to 79% achieved Phenylalanine ≤ 120 μmol/L after 24 months	Injection site reactions, Arthralgia, headache, risk of anaphylaxis (3.8%); not tested in pregnancy	Food and Drug Administration–approved (2018) for adults	Nulmans et al., 2025

## 7 Limitations and challenges

Reduction in PAH activity leads to various changes in the synthesis of cerebral myelin and proteins. Additionally, the levels of serotonin, dopamine, and noradrenaline in the brain are significantly reduced. If left untreated, the development of the brain is extremely affected, causing significant intellectual disability and behavioral interference. Currently, only a few methods are involved in overcoming the challenges faced by Phenylketonuria. The use of the neonatal heel prick diagnostic method during birth can be used to analyze diseases at an earlier stage. The inclusion of a proper phenylalanine-confined diet (dietary treatment) has led to the maintenance of adequate neurodevelopment until adulthood. Studies state that the dietary method notably improved cognitive and psychiatric levels of the Phenylketonuria-affected individuals. Nevertheless, few individuals who maintain their nutritional habits persistently with proper control also have an increasing risk of mood, anxiety, or attentional disorders throughout their lives. On the other hand, the resulting effects of prolonged protein-limited foods on the functioning of the brain remain unexplored. Further, a concentration of 120–360 μmol/L blood phenylalanine should be maintained throughout the lifespan. The availability of limited data for clinicians makes it challenging to treat adult Phenylketonuria patients with psychiatric disorders. Further, advancements in 3D food printing are emerging for personally curated, high-nutrient-optimized, and sensory-appetizing foods, providing a novel dietary therapy for Phenylketonuria patients. In addition, a novel treatment like enzyme therapy holds promising results without limited dietary practices for better cognitive processing and mental health. For instance, regular subcutaneous injection of pegylated Phenylalanine ammonia-lyase or pegvaliase (PALYNZIQ^®^) serves as a propitious genetic therapy in the clinical trials done recently. Casein glycomacropeptide improves immunological processes and enhances large neutral amino acids for the prevention of plasma Phe transportation to the brain. Sapropterin hydrochloride or Kuvan^®^, an analog of BH4, aids as a potential therapy for activating residual PAH, which in turn decreases the blood phenylalanine level. Though these therapies help in managing the disease, the identification of new Phenylketonuria modifiers is necessary in the future. Other options, like cost-effective next-generation sequencing, can be used to rapidly analyze the genes and genomes to provide personalized diagnostics in the future (Russo et al., 2025; Ashe et al., 2019; Elhawary et al., 2022).

## 8 Future perspectives

Emerging research on the gut microbiota’s role in phenylalanine metabolism opens new avenues for microbiome-targeted therapies in Phenylketonuria management. Future strategies may include personalized probiotics or engineered gut microbes to modulate Phenylalanine levels and reduce toxic metabolites like PAGln. Further studies on microbial pathways could aid in discovering novel bioactive compounds impacting systemic health. Advances in precision nutrition and microbiota editing may improve cognitive outcomes in Phenylketonuria patients. Long-term clinical trials are essential to evaluate the safety, efficacy, and sustainability of these innovative microbiome-based interventions.
